# Supplementary material for: Repurposing conformational changes in ANL superfamily enzymes to rapidly generate biosensors for organic and amino acids
Source: Nat Commun. 2023 Oct 21;14:6680. doi: 10.1038/s41467-023-42431-y (PMC10590383; doi:10.1038/s41467-023-42431-y)
Supplement: Supplementary file 1 — Supplementary Information [file 41467_2023_42431_MOESM1_ESM.pdf]

Repurposing conformational changes in ANL superfamily enzymes to rapidly generate biosensors for organic and amino acids

Wang *et al.*

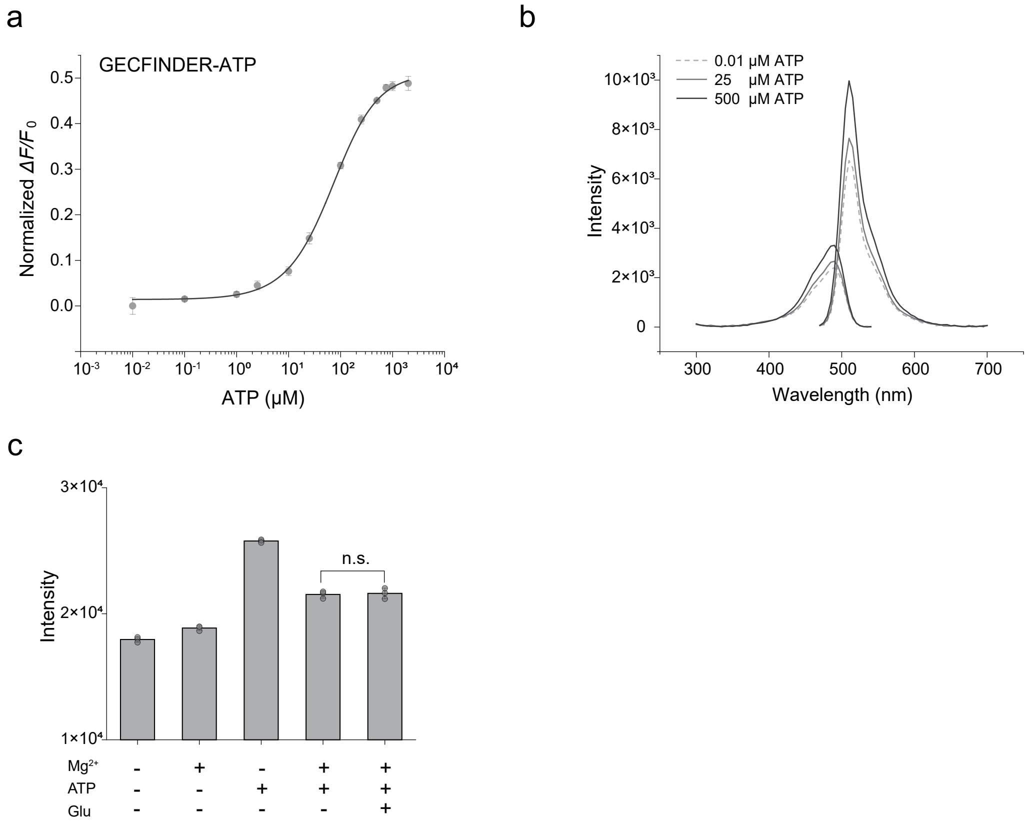

Supplementary Fig. 1 **Dose response curves and fluorescence spectrums of GECFINDER-ATP. a,** Dose–response curve of purified GECFINDER-ATP with increasing concentrations of ATP (10 nM to 2 mM). The normalized $\Delta F/F_0$ value increased after ATP binding (480 nm excitation). **b,** The fluorescence spectra of GECFINDER-ATP changed as the ATP concentration increased from 10 nM to 500 µM. **c**, GECFINDER-ATP did not respond to glutamate. All data shown are means ± S.D. (n=3 biologically independent samples). *p ≤ 0.05, ***p ≤ 0.001, ****p ≤ 0.0001, and n.s. indicated no significant difference (Student's two-tailed t-test). Source data are provided as a Source Data file.

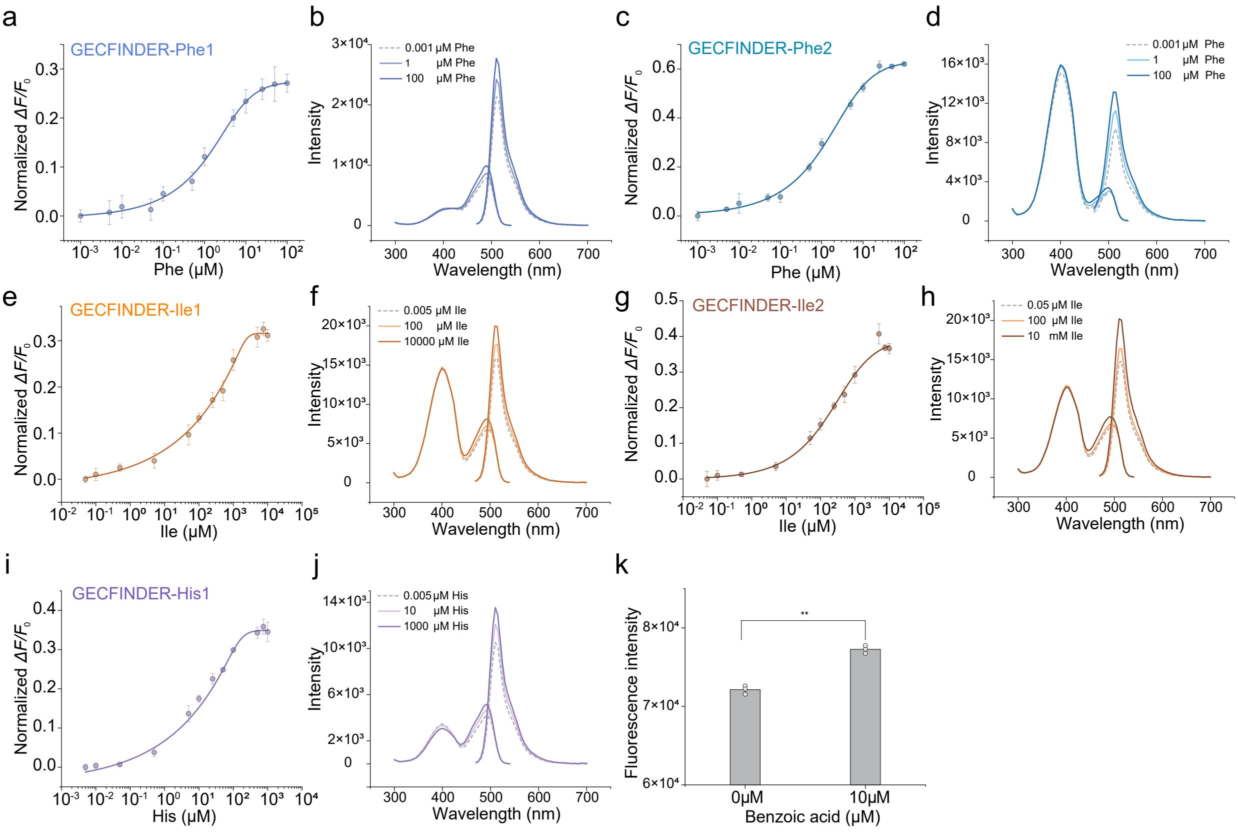

Supplementary Fig. 2 **Dose–response curves and fluorescence spectrums of GECFINDERs. a and c,** Dose–response curves of purified GECFINDER-Phe1 and 2 with increasing concentrations of phenylalanine (1 nM to 100 μM). The normalized $\Delta F/F_0$ value increased after phenylalanine binding (460 nm excitation). **b and d,** The fluorescence spectra of GECFINDER-Phe1 and 2 changed as the phenylalanine concentration increased from 1 nM to 100 μM. **e and g,** Dose–response curves of purified GECFINDER-Ile1 and 2 with increasing concentrations of isoleucine (50 nM to 10 mM). The normalized $\Delta F/F_0$ value increased after isoleucine binding (480 nm excitation). **f and h,** The fluorescence spectra of GECFINDER-Ile1 and 2 changed as the isoleucine concentration increased from 0.5 μM to 10 mM. **i,** Dose–response curve of purified GECFINDER-His1 with increasing concentrations of histidine (5 nM to 1 mM). The Normalized $\Delta F/F_0$ increased after histidine binding (480 nm excitation). **j,** The fluorescence spectra of GECFINDER-His1 changed as the histidine concentration increased from 5 nM to 1 mM. **k,** Difference in fluorescence intensity of GECFINDER-benzoic acid in the presence of 10 μM benzoic acid compared to the control (0 μM benzoic acid). All data shown are means ± S.D. (n=3 biologically independent samples). $^*p \leq 0.05$, $^{***}p \leq 0.001$, $^{****}p \leq 0.0001$, and n.s. indicated no significant difference (Student's two-tailed t-test). Source data are provided as a Source Data file.

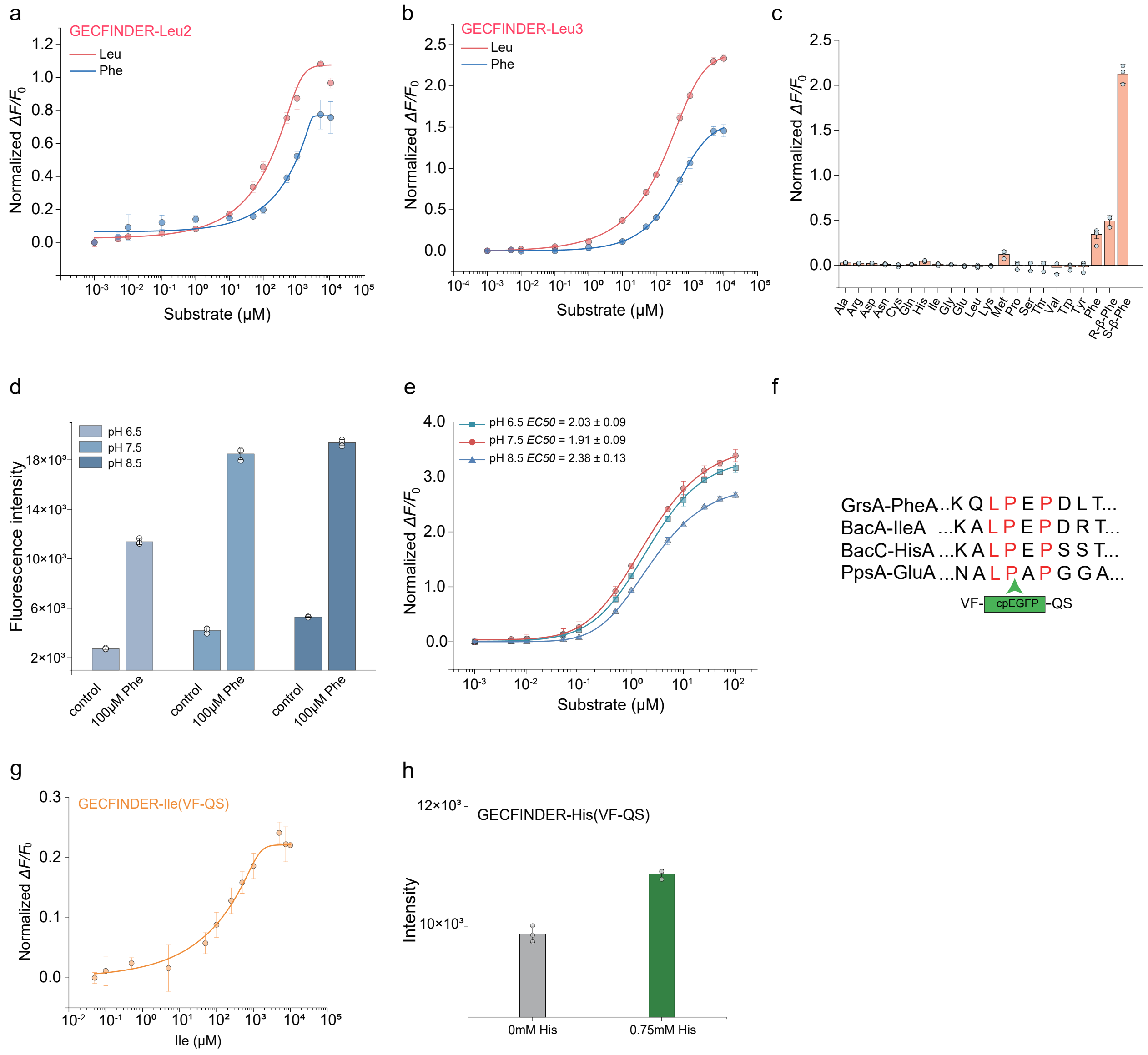

Supplementary Fig. 3 **Substrate specificity engineering and cpEGFP insertion site optimization of GECFINDER. a and b,** Dose–response curves of purified GECFINDER-Leu2 and 3with increasing concentrations of Leu and Phe. **c,** The normalized $\Delta F/F_0$ value of GECFINDER-SβF with 20 proteinogenic amino acids and R/S-β-Phe. The concentrations of all kinds of substrates were 100 μM. **d,** Fluorescence intensity of purified GECFINDER-Phe3 when incubated with phenylalanine at different pH levels, control was the fluorescence intensity when no substrate was added. **e,** Dose – response curves of purified GECFINDER-Phe3 with increasing concentrations of Phe at different pH levels. **f,** The LPXP motif at the junction between the end of the A domain and the PCP domain. **g,** Dose–response curve of purified GECFINDER-Ile (VF-QS) with increasing concentrations of isoleucine (50 nM to 10 mM). The Normalized $\Delta F/F_0$ value increased after isoleucine binding (480 nm excitation).**h,** Difference in fluorescence intensity of GECFINDER-His (VF-QS) in the presence of 0.75 mM histidine compared to the control (0 mM histidine). All data shown are means ± S.D. (n=3 biologically independent samples). Source data are provided as a Source Data file.

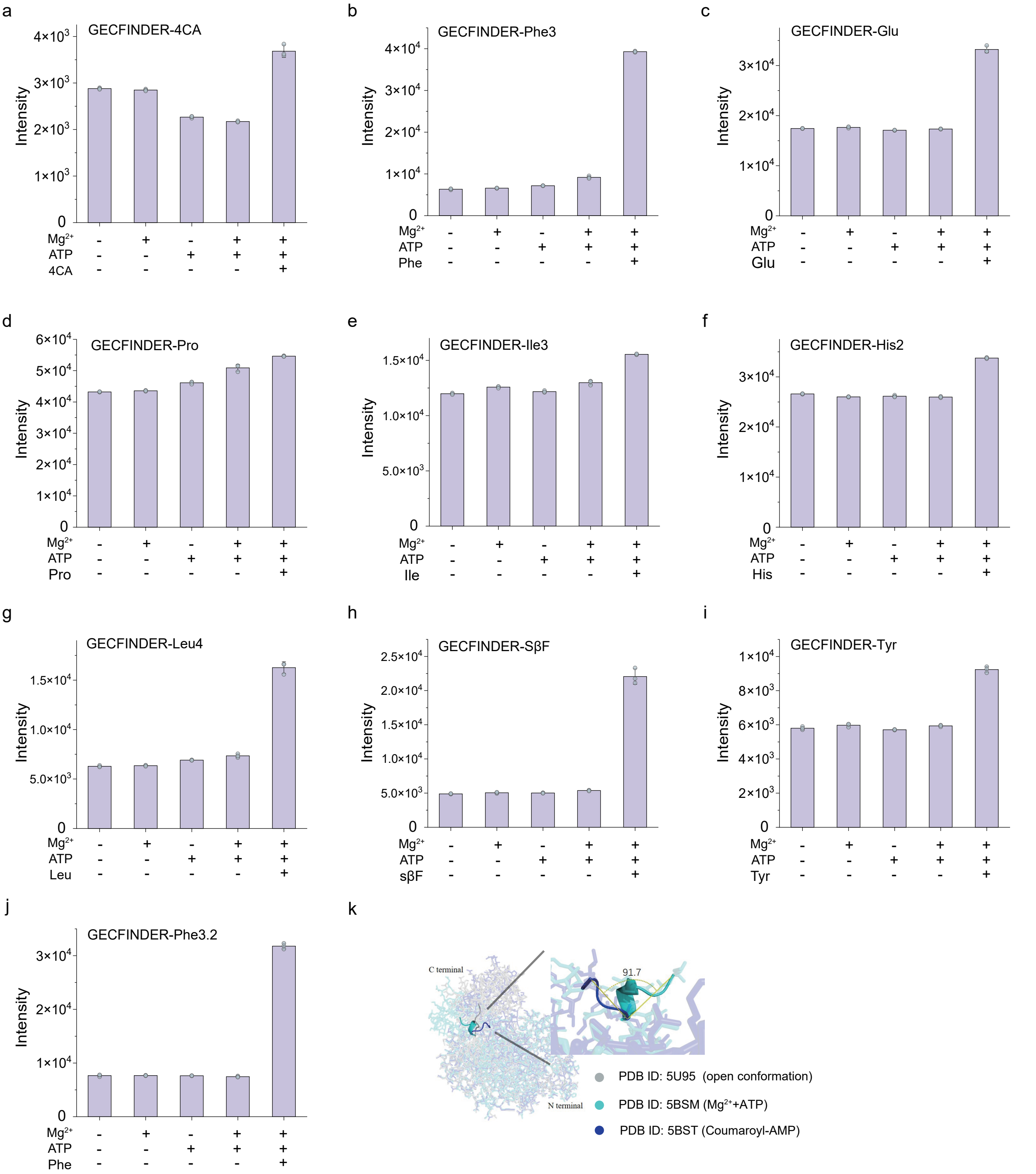

Supplementary Fig. 4 **Dependence of GECFINDERs on Mg^{2+} and ATP. a-j**, Fluorescence intensities of GECFINDERs in the absence of any substrate, in the presence of Mg^{2+} alone, ATP alone, both Mg^{2+} and ATP, Mg^{2+} and ATP with respective substrates. Mg^{2+} concentration was 2.5 mM, and ATP concentration was 1 mM. **a**, 100 μM 4-coumaric acid, **b**, 100 μM phenylalanine, **c**, 1 mM glutamine, **d**, 1 mM proline, **e**, 100 μM isoleucine, **f**, 1 mM histidine, **g**, 1 mM leucine, **h**, 1 mM S-β-phenylalanine, **i**, 1 mM tyrosine, **j**, 1 mM phenylalanine. **k**, Conformational change of Nt4CL2 at Hinge A. All data shown are means ± S.D. (n=3 biologically independent samples). Source data are provided as a Source Data file.

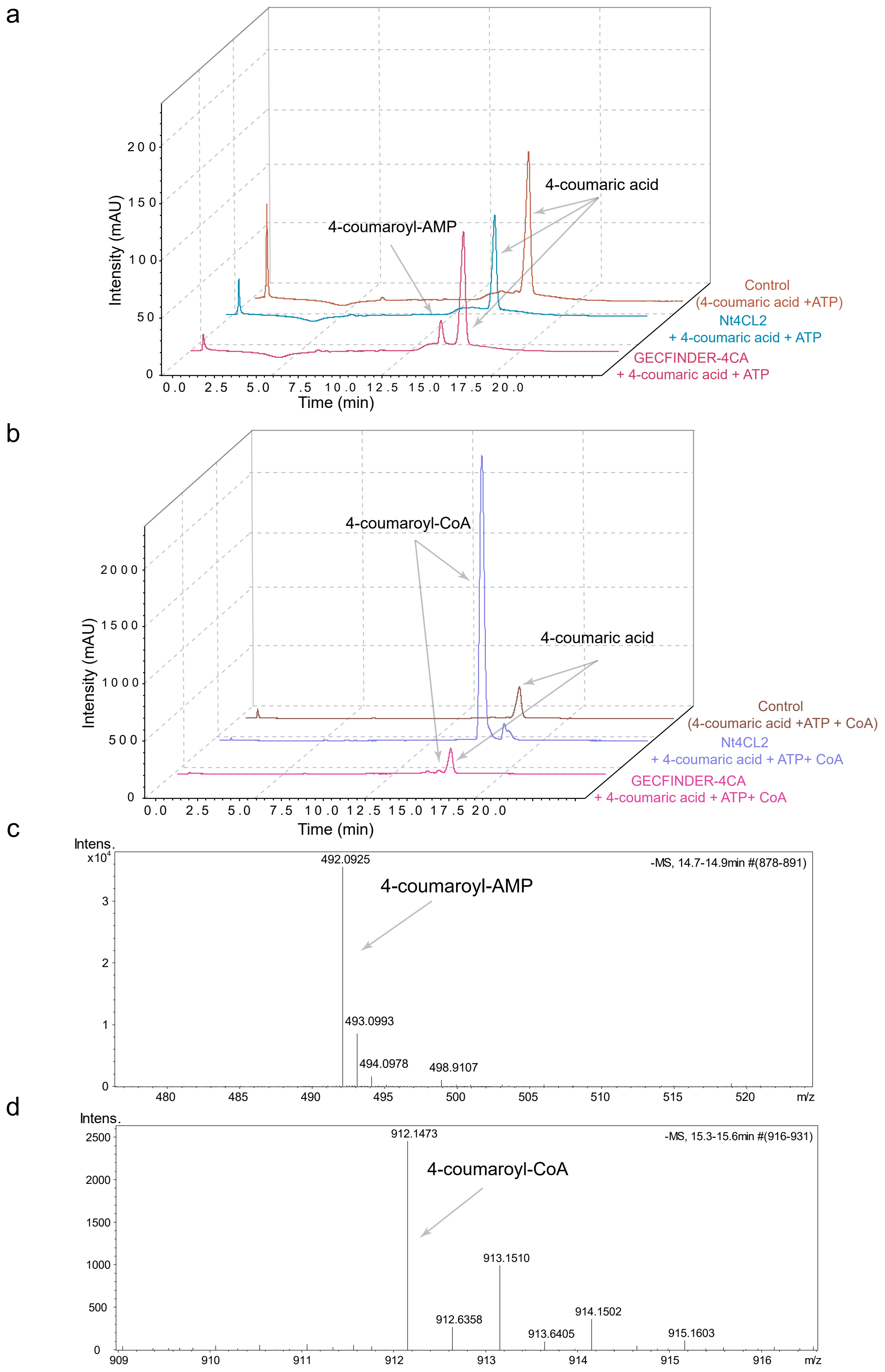
a
Intensity (mAU)
0
50
100
150
200
0.0 2.5 5.0 7.5 10.0 12.5 15.0 17.5 20.0
Time (min)
4-coumaroyl-AMP
4-coumaric acid
Control
(4-coumaric acid +ATP)
Nt4CL2
+ 4-coumaric acid + ATP
GECFINDER-4CA
+ 4-coumaric acid + ATP
b
Intensity (mAU)
0
500
1000
1500
2000
0.0 2.5 5.0 7.5 10.0 12.5 15.0 17.5 20.0
Time (min)
4-coumaroyl-CoA
4-coumaric acid
Control
(4-coumaric acid +ATP + CoA)
Nt4CL2
+ 4-coumaric acid + ATP+ CoA
GECFINDER-4CA
+ 4-coumaric acid + ATP+ CoA
c
Intens.
x10^4
-MS, 14.7-14.9min #(878-891)
492.0925
4-coumaroyl-AMP
493.0993
494.0978
498.9107
0
1
2
3
480 485 490 495 500 505 510 515 520 m/z
d
Intens.
-MS, 15.3-15.6min #(916-931)
912.1473
4-coumaroyl-CoA
913.1510
912.6358
913.6405
914.1502
915.1603
0
500
1000
1500
2000
2500
909 910 911 912 913 914 915 916 m/z

Supplementary Fig. 5 **Influence of cpEGFP insertion on the catalytic performance of GECFINDER-4CA a,** Chromatographic analysis of GECFINDER-4CA, Nt4CL2 and control reaction intermediates in the presence of ATP and coumaric acid after 24 hours. The traces shown are monitored at 340 nm. **b,** Chromatographic analysis of GECFINDER-4CA, Nt4CL2 and control reaction products in the presence of ATP, coumaric acid and CoA after 24 hours. The traces shown are monitored at 333 nm. **c and d,** Mass spectra of the intermediate and thioester product formed by GECFINDER-4CA.

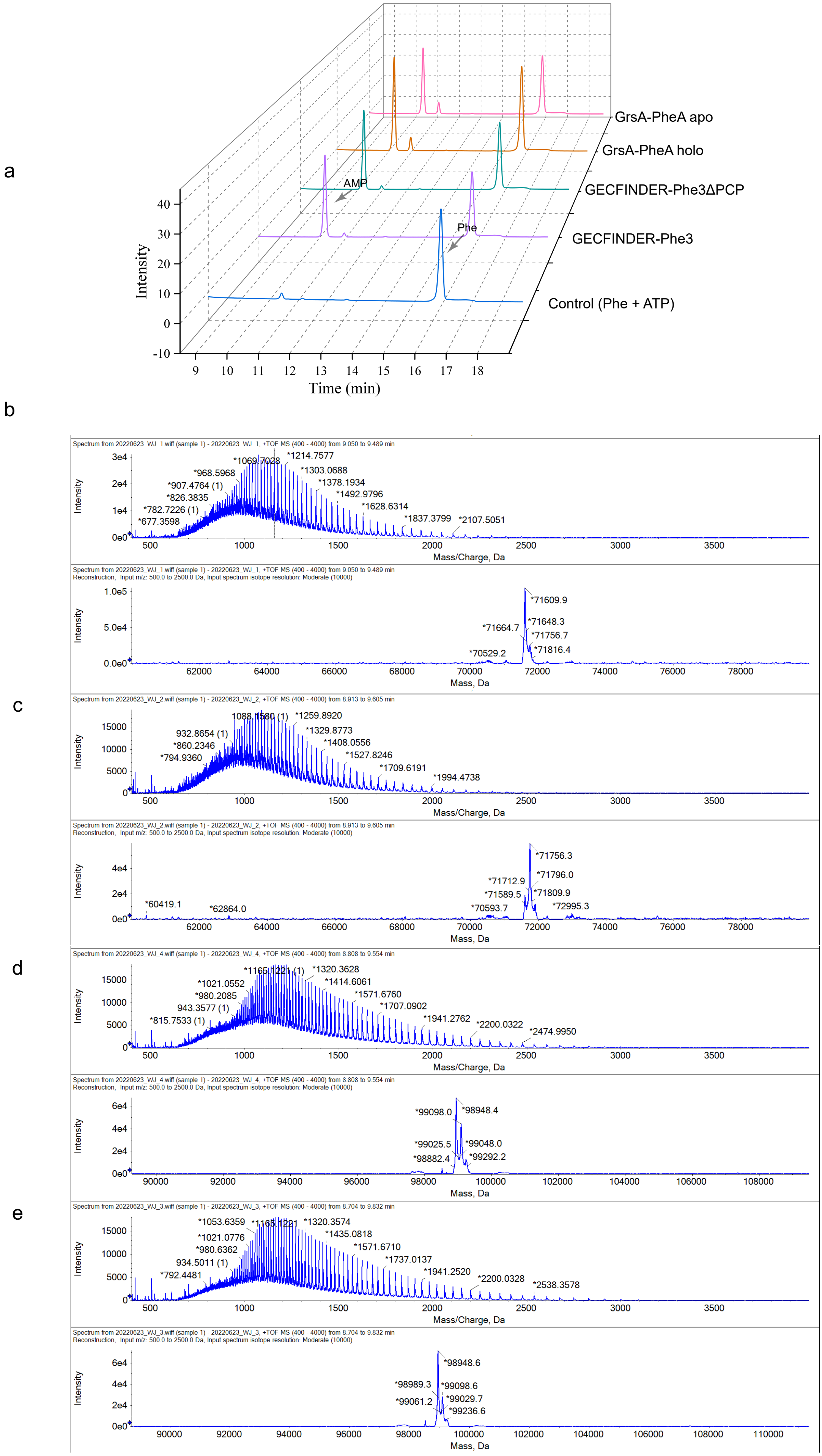

a
GrsA-PheA apo
GrsA-PheA holo
GECFINDER-Phe3ΔPCP
GECFINDER-Phe3
Control (Phe + ATP)
AMP
Phe
Intensity
Time (min)
b
c
d
e
Spectrum from 20220623_WJ_1.wiff (sample 1) - 20220623_WJ_1, +TOF MS (400 - 4000) from 9.050 to 9.489 min
Reconstruction, Input m/z: 500.0 to 2500.0 Da, Input spectrum isotope resolution: Moderate (10000)
Spectrum from 20220623_WJ_2.wiff (sample 1) - 20220623_WJ_2, +TOF MS (400 - 4000) from 8.913 to 9.605 min
Spectrum from 20220623_WJ_4.wiff (sample 1) - 20220623_WJ_4, +TOF MS (400 - 4000) from 8.808 to 9.554 min
Spectrum from 20220623_WJ_3.wiff (sample 1) - 20220623_WJ_3, +TOF MS (400 - 4000) from 8.704 to 9.832 min
Mass/Charge, Da
Mass, Da
*71609.9
*71756.3
*98948.4
*98948.6

Supplementary Fig. 6 **Influence of cpEGFP insertion on the catalytic performance of GECFINDER-Phe3 a,** Representative HPLC traces for 7 μM GECFINDER-Phe3, GECFINDER-Phe3ΔPCP, and GrsA-PheA holo/apo with 1 mM Phe and 1 mM ATP. The traces shown are monitored at 254 nm. **b and c,** Representative raw mass data for GrsA-PheA holo without (b) and with ATP and Phe (c). **d and e,** Representative raw mass data for GECFINDER-Phe3 without (d) and with ATP and Phe (e).

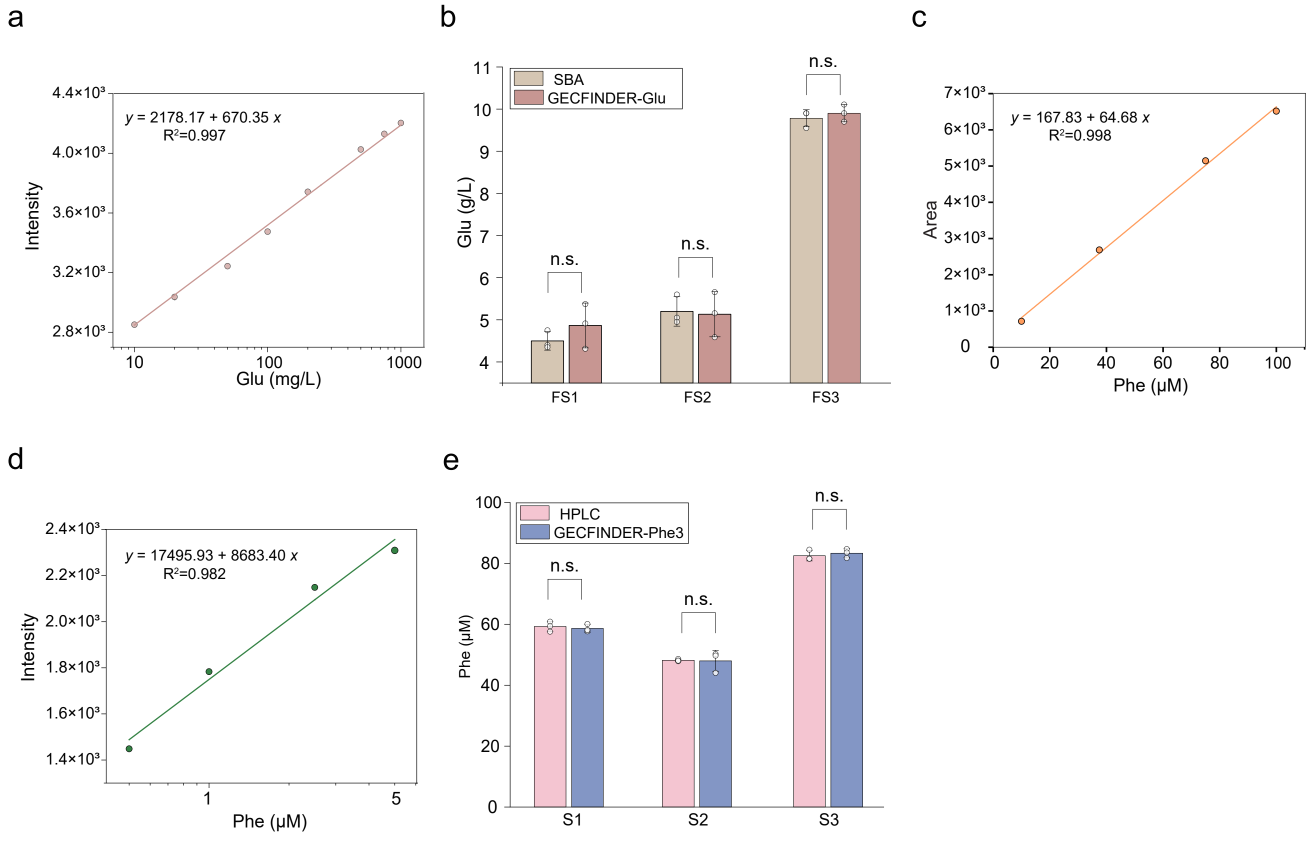

Supplementary Fig. 7 **Accurate quantification of amino acid concentrations in biological samples with GECFINDERs. a,** Linear dependence between GECFINDER-Glu fluorescence intensity and Glu concentrations. The equation is $y = 2178.17 + 670.35\ x$, where the fitting slope is used to convert GECFINDER-Glu fluorescence intensity to Glu concentrations. **b,** Concentration of Glu in the fermentation broth samples determined by both SBA and GECFINDER-Glu methods. **c,** Linear dependence between Phe peak areas and Phe concentrations. The equation is $y = 167.83 + 64.68\ x$, where the fitting slope is used to convert Phe peak areas to Phe concentrations. **d,** Linear dependence between GECFINDER-Phe3 fluorescence intensity and Phe concentrations. The equation is $y = 17495.93 + 8683.40\ x$, where the fitting slope is used to convert GECFINDER-Phe3 fluorescence intensity to Phe concentrations. **e,** Concentration of phenylalanine in the blood samples determined by both GECFINDER-Phe3 and HPLC methods. All data shown are means ± S.D. (n=3 biologically independent samples). *$p \leq 0.05$, ***$p \leq 0.001$, ****$p \leq 0.0001$, and n.s. indicated no significant difference (Student's two-tailed t-test). Source data are provided as a Source Data file.

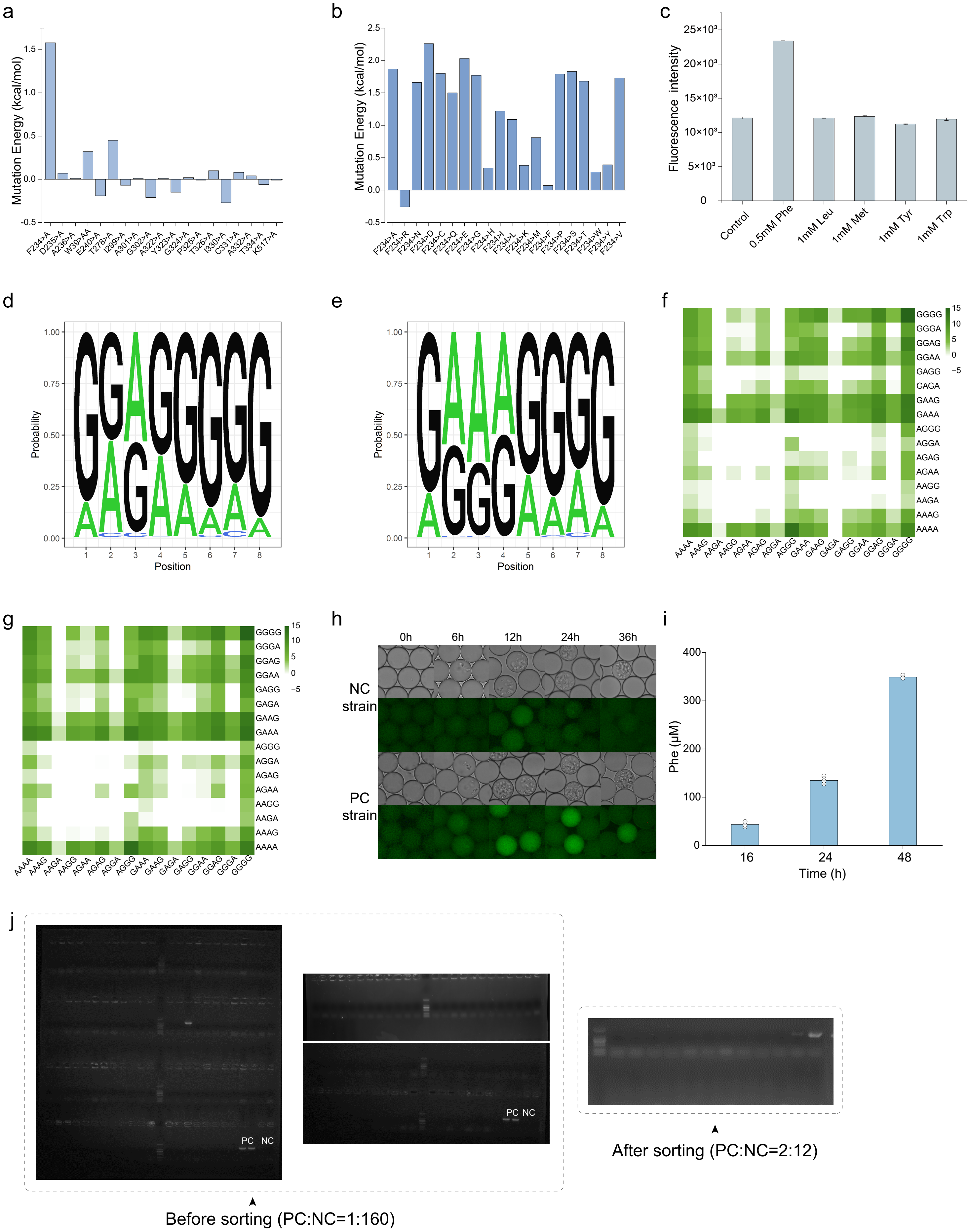

a
Mutation Energy (kcal/mol)
1.5
1.0
0.5
0.0
-0.5
F234>A
D235>A
A236>A
W39>AA
E240>A
T278>A
I299>A
A301>A
G302>A
A322>A
Y323>A
G324>A
P325>A
T326>A
I330>A
C331>A
A332>A
T334>A
K517>A
b
Mutation Energy (kcal/mol)
2.5
2.0
1.5
1.0
0.5
0.0
-0.5
F234>A
F234>R
F234>N
F234>D
F234>C
F234>Q
F234>E
F234>G
F234>H
F234>I
F234>L
F234>K
F234>M
F234>F
F234>P
F234>S
F234>T
F234>W
F234>Y
F234>V
c
Fluorescence intensity
25×10³
20×10³
15×10³
10×10³
5×10³
0
Control
0.5mM Phe
1mM Leu
1mM Met
1mM Tyr
1mM Trp
d
Probability
1.00
0.75
0.50
0.25
0.00
1
2
3
4
5
6
7
8
Position
e
Probability
1.00
0.75
0.50
0.25
0.00
1
2
3
4
5
6
7
8
Position
f
GGGG
GGGA
GGAG
GGAA
GAGG
GAGA
GAAG
GAAA
AGGG
AGGA
AGAG
AGAA
AAGG
AAGA
AAAG
AAAA
15
10
5
0
−5
AAAA
AAAG
AAGA
AAGG
AGAA
AGAG
AGGA
AGGG
GAAA
GAAG
GAGA
GAGG
GGAA
GGAG
GGGA
GGGG
g
GGGG
GGGA
GGAG
GGAA
GAGG
GAGA
GAAG
GAAA
AGGG
AGGA
AGAG
AGAA
AAGG
AAGA
AAAG
AAAA
15
10
5
0
−5
AAAA
AAAG
AAGA
AAGG
AGAA
AGAG
AGGA
AGGG
GAAA
GAAG
GAGA
GAGG
GGAA
GGAG
GGGA
GGGG
h
0h
6h
12h
24h
36h
NC strain
PC strain
i
Phe (μM)
400
300
200
100
0
16
24
48
Time (h)
j
PC NC
PC NC
Before sorting (PC:NC=1:160)
After sorting (PC:NC=2:12)

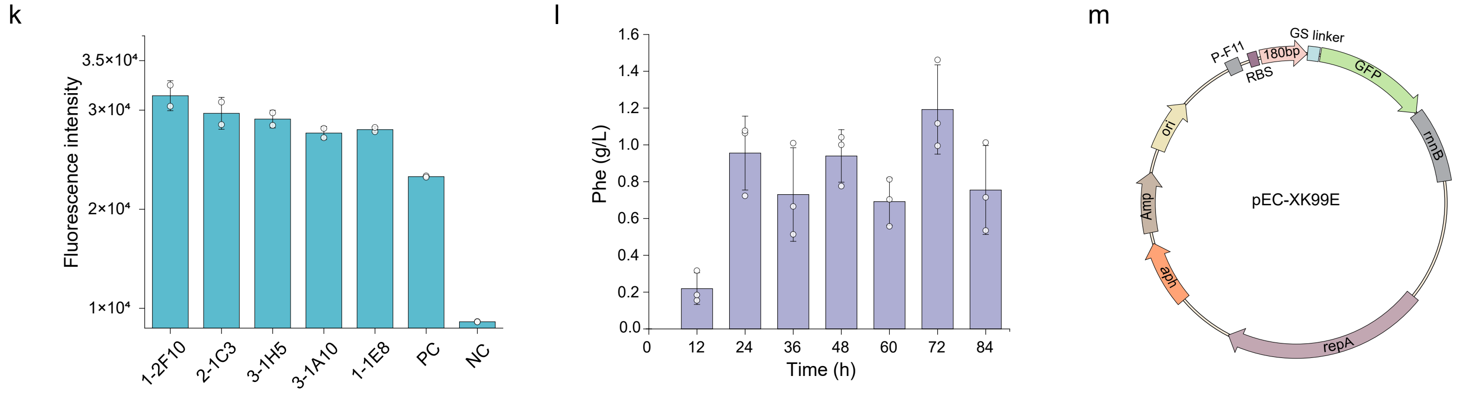

Supplementary Fig. 8 **GECFINDER-Phe3 engineering and applying for FDAS sorting. a,** The mutation energies obtained by alanine scan of the substrate binding site of GrsA-PheA using Discovery studio 2019. **b,** The mutation energies obtained by virtual saturation mutation of F234 of GrsA-PheA using Discovery studio 2019. **c**, The fluorescence intensity when GECFINDER-Phe3.2 was incubated with different substrates, control was the fluorescence intensity when no substrate was added. Data shown are means ± S.D. (n=3 biologically independent samples). **d,** Components of RBS library of *pheA* ^{fbr} generated by BETTER. **e,** Components of RBS library of *aroF* ^{fbr} generated by BETTER. **f**, The matrix showed the coverage of 256 G/A-containing RBS variants in the *pheA*^{fbr} RBS library generated by BETTER. **g**, The matrix showed the coverage of 256 G/A-containing RBS variants in the *aroF*^{fbr} RBS library generated by BETTER. **h**, Microscopic observation of NC and PC strains droplets at different time intervals. Scale bar: 50μm. **i,** The phenylalanine production of PC strain after 16, 24 and 48 hours of static culture. Data shown are means ± S.D. (n=3 biologically independent samples). **j,** PC strain ratio verification by colony PCR and agarose gel electrophoresis. **k,** The fluorescence intensity of five phenylalanine high yielding strains screened with GECFINDER-Phe3. Data shown are means ± S.D. (n=2 biologically independent samples) **l**, The phenylalanine production of 1-2 F10 in shake flask fermentation in CGXII at different time periods. Data shown are means ± S.D. (n=3 biologically independent samples).**m,** Components of the RBS strength reporter system. Source data are provided as a Source Data file.

Supplementary Table 1. The thermal stability of GECFINDER-Phe.

Biosensor	T_{m1}	T_{onset}	T_{m2}	T_{agg266}	T_{agg473}
GECFINDER-Phe3	48.16±0.12	45.46±1.43	67.93±0.41	52.73±0.93	56.42±1.58
GECFINDER-Phe3.2	49.00±0.11	44.85±1.73	68.91±0.81	52.49±1.56	56.85±1.56

Supplementary Table 2. The absolute fluorescence quantum yields of GECFINDERs.

Biosensor	Ligand	λ_{ex}(nm)	λ_{em}(nm)	φ[a]	
				Apo	Sat
GECFINDER-4CA	4-coumaric acid	400	510	0.18	0.54
GECFINDER-Phe3	Phe	460	510	0.15	0.47
GECFINDER-Phe3.2	Phe	460	510	0.15	0.46
GECFINDER-Glu	Glu	480	510	0.41	0.56
GECFINDER-Pro	Pro	480	510	0.37	0.44
GECFINDER-Ile3	Ile	480	510	0.08	0.52
GECFINDER-His2	His	480	510	0.19	0.56
GECFINDER-Leu4	Leu	480	510	0.08	0.36
GECFINDER-SβF	S-β-Phe	460	510	0.32	0.37
GECFINDER-Tyr	Tyr	480	510	0.10	0.38

[a], Quantum Yield in the absence (Apo) or presence (Sat) of the corresponding analyte (4-coumaric acid, 100 µM. Phe, 100 µM for GECFINDER-Phe3, 1mM for GECFINDER-Phe3.2. Glu, 1mM. Pro, 1mM. Ile, 1mM. His, 1mM. Leu, 1mM. S-β-Phe, 1mM. Tyr, 1mM.).

Supplementary Table 3. The information about the gender and age of the research participants.		
**Participants**	**Gender**	**Age**
Participants 1	Female	29
Participants 2	Male	27
Participants 3	Female	24

Supplementary Note 1

As a distant relative of the ANL superfamily, carboxylic acid reductases (CARs) harbor domains like NRPSs and can reduce carboxylic acids to the corresponding aldehydes using ATP and NADPH as cofactors, undergoing significant substrate-mediated conformational changes like the ANL superfamily enzymes[1]. CARs can be classified into four classes (α-aminoadipic acid reductase, aryl acid reductase, tyrosine reductase, and serine reductase) and have a wide range of substrates including aromatic, heteroaromatic, and aliphatic derivatives[2], which can also be used as LBDs for GECFINDER. Using a similar screening method, we obtained an effective biosensor that used the A and PCP domains of SrCAR from *Segniliparaceae rugosus* as an LBD and benzoic acid as a corresponding ligand. This biosensor exhibited a relatively low dynamic range; the fluorescence intensity increased by 7.1 ± 0.01% compared with the control in the presence of benzoic acid (Supplementary Fig. 2 k).

One of the less efficient aspects of the current GECFINDER creation process is that it still requires screening, albeit limited, to identify the functional insertion site and efficient linkers. Therefore, we intended to rationally design the effective insertion site of cpEGFP. In about 70% of the NRPS A domain sequences, there is a relatively conserved LPXP motif in the junction between the end of the A and the PCP domain[3], and the successful cpEGFP insertion site of GECFINDER-Phe1–3 is in the middle of the LPXP motif. We speculated that the LPXP motif was a promising insertion site for creating effective GECFINDERs. Therefore, cpEGFP with a fixed linker (the same as GECFINDER-Phe3: VF-cpEGFP-QS) was inserted into the middle of the LPXP motif

in the LBD of GECFINDER-Ile/His/Glu (Supplementary Fig. 3 f). We found that insertion into the LPXP motif of GECFINDER-Ile exhibited the best performance for the linker VF-cpEGFP-QS among the three LBDs, where the $\Delta F/F_0$ value reached 0.22 ± 0.003 (Supplementary Fig. 3 g). The LBD of GECFINDER-His (VF-QS) represented a narrower dynamic range for the linker VF-cpEGFP-QS, where the $\Delta F/F_0$ value was 0.1 ± 0.01 (Supplementary Fig. 3 h). However, insertion into the LPXP motif of GECFINDER-Glu with the linker VF-cpEGFP-QS did not generate an active glutamate biosensor. In conclusion, although the dynamic range was not wide, three quarters of the tested LBDs with the LPXP motif were compatible with the fixed linker VF-cpEGFP-QS, indicating that the LPXP motif is indeed a promising engineering site to construct functional GECFINDERs as well as a preferable cpEGFP insertion site for GECFINDER screening.

Supplementary References

1. Qu G*, et al.* Computational Insights into the Catalytic Mechanism of Bacterial Carboxylic Acid Reductase. *J Chem Inf Model* **59**, 832-841 (2019).
2. Qu G, Guo J, Yang D, Sun Z. Biocatalysis of carboxylic acid reductases: phylogenesis, catalytic mechanism and potential applications. *Green Chem.* **20**, 777-792 (2018).
3. Miller BR, Sundlov JA, Drake EJ, Makin TA, Gulick AM. Analysis of the linker region joining the adenylation and carrier protein domains of the modular nonribosomal peptide synthetases. *Proteins* **82**, 2691-2702 (2014).
