# Supplementary material for: Repurposing conformational changes in ANL superfamily enzymes to rapidly generate biosensors for organic and amino acids
Source: Nat Commun. 2023 Oct 21;14:6680. doi: 10.1038/s41467-023-42431-y (PMC10590383; doi:10.1038/s41467-023-42431-y)
Supplement: Supplementary file 3 — Reporting Summary [file 41467_2023_42431_MOESM3_ESM.pdf]

nature portfolio

Corresponding author(s): Meng, W.

Last updated by author(s): Oct 6, 2023

Reporting Summary

Nature Portfolio wishes to improve the reproducibility of the work that we publish. This form provides structure for consistency and transparency in reporting. For further information on Nature Portfolio policies, see our Editorial Policies and the Editorial Policy Checklist.

Statistics

For all statistical analyses, confirm that the following items are present in the figure legend, table legend, main text, or Methods section.

n/a	Confirmed	
☐	☒	The exact sample size (*n*) for each experimental group/condition, given as a discrete number and unit of measurement
☐	☒	A statement on whether measurements were taken from distinct samples or whether the same sample was measured repeatedly
☐	☒	The statistical test(s) used AND whether they are one- or two-sided<br>*Only common tests should be described solely by name; describe more complex techniques in the Methods section.*
☒	☐	A description of all covariates tested
☒	☐	A description of any assumptions or corrections, such as tests of normality and adjustment for multiple comparisons
☐	☒	A full description of the statistical parameters including central tendency (e.g. means) or other basic estimates (e.g. regression coefficient) AND variation (e.g. standard deviation) or associated estimates of uncertainty (e.g. confidence intervals)
☐	☒	For null hypothesis testing, the test statistic (e.g. *F*, *t*, *r*) with confidence intervals, effect sizes, degrees of freedom and *P* value noted<br>*Give P values as exact values whenever suitable.*
☒	☐	For Bayesian analysis, information on the choice of priors and Markov chain Monte Carlo settings
☒	☐	For hierarchical and complex designs, identification of the appropriate level for tests and full reporting of outcomes
☒	☐	Estimates of effect sizes (e.g. Cohen's *d*, Pearson's *r*), indicating how they were calculated

Our web collection on statistics for biologists contains articles on many of the points above.

Software and code

Policy information about availability of computer code

Data collection	1.Biotek Gen5 v2.04 (microplate reader software)<br>2.Discovery Studio 2019<br>3. OpenLAB CDS ChemStation Edition for Agilent 1260 Infinity Ⅱ HPLC system (Agilent, Waldbronn, Germany) was used for collection of HPLC data.<br>4. UNIFI (v 1.9.4.053, Waters, Corp.) for Q-TOF MS (Waters, Wilmslow, UK) was used for collection of LC-MS data.<br>4. Analyst TF (v 1.8.1) for TripleTOF 6600 (SCIEX, Massachusetts state, USA) was used for collection of MS data.<br>3. Illumina Hiseq Control software was used on the Illumina Hiseq sequencers to collect the sequencing data.
Data analysis	1. All P values were generated from two-tailed t-tests using the Microsoft Excel 2019.<br>2. The dose-response curves were fitted using the logistic model LOGISTIC5 available in Origin 2021.<br>3. Linear fit of the standard curve and sample concentration calculation were performed using Origin 2021 software.<br>4. For NGS data analysis, 20-nT sequences upstream of the target region were used to locate the position of the target region in each read by BLAST. The sequence of the target region was then extracted from the reads and mapped to a reference sequence to analyze base editing events. Each RBS variant was quantified using the R package (v4.0.2). Sequence logos were generated using the statistics of the RBS variants containing G/A/C/T and the R package 'ggseqloo' (v0.1). Heat maps were generated using the statistics of RBS variants containing G/A and the R package 'ggplot2' (v3.3.2).

For manuscripts utilizing custom algorithms or software that are central to the research but not yet described in published literature, software must be made available to editors and reviewers. We strongly encourage code deposition in a community repository (e.g. GitHub). See the Nature Portfolio guidelines for submitting code & software for further information.

Data

Policy information about availability of data

All manuscripts must include a data availability statement. This statement should provide the following information, where applicable:

- Accession codes, unique identifiers, or web links for publicly available datasets
- A description of any restrictions on data availability
- For clinical datasets or third party data, please ensure that the statement adheres to our policy

The data supporting the findings of this work are available within the paper and the Supplementary Information files. The protein structure data used this study are available in the RCSB PDB under accession codes 1AMU [https://www.rcsb.org/structure/1AMU], 5U95 [https://www.rcsb.org/structure/5U95], 5BSM [https://www.rcsb.org/structure/5BSM] and 5BST [https://www.rcsb.org/structure/5BST]. The raw reads of the NGS data were deposited into the Sequence Read Archive database of NCBI under accession number PRJNA1019141 [https://www.ncbi.nlm.nih.gov/bioproject/PRJNA1019141] . Source data are provided with this paper.

Human research participants

Policy information about studies involving human research participants and Sex and Gender in Research.

Reporting on sex and gender	All analysis pertained to people of both sexes, information on which was self-reported. No separate sex-based analyses were performed due to the lack of statistical power.
Population characteristics	This study did not collect any information regarding the health status of the participants. The average age of the participants was 27 years old.
Recruitment	The study participants were recruited from within the laboratory's personnel. Interested individuals received an Informed Consent Form (ICF), during which researchers explained the ICF to the participants. Volunteers who chose to participate signed the ICF voluntarily. While there was a possibility of self-selection bias, this was unlikely to affect the outcome due to the dependence on physical measures. No compensation was provided to participants in this study.
Ethics oversight	Ethics Committee of Tianjin Institute of Industrial Biotechnology, Chinese Academy of Sciences (TIB202306-002).

Note that full information on the approval of the study protocol must also be provided in the manuscript.

Field-specific reporting

Please select the one below that is the best fit for your research. If you are not sure, read the appropriate sections before making your selection.

☒ Life sciences ☐ Behavioural & social sciences ☐ Ecological, evolutionary & environmental sciences

For a reference copy of the document with all sections, see nature.com/documents/nr-reporting-summary-flat.pdf

Life sciences study design

All studies must disclose on these points even when the disclosure is negative.

Sample size	All data is reported as triplicate measurements unless stated differently. All individual data points are shown in figures, and total sample size spanning all independent experiments is reported in the figure captions.
Data exclusions	No data was excluded from the analysis.
Replication	The replication number of each data is indicated in the legend of the corresponding figures. All attempts at replication were successful.
Randomization	While randomization is not relevant to this study since our biochemical reactions are handled uniformly, the same data analysis procedure was applied to all samples of the same type.
Blinding	Blinding is not relevant to this study since the results presented are based on objective description of our study, and are therefore not subject to human biases.

Reporting for specific materials, systems and methods

We require information from authors about some types of materials, experimental systems and methods used in many studies. Here, indicate whether each material, system or method listed is relevant to your study. If you are not sure if a list item applies to your research, read the appropriate section before selecting a response.

Materials & experimental systems

n/a	Involved in the study	
☒	☐	Antibodies
☒	☐	Eukaryotic cell lines
☒	☐	Palaeontology and archaeology
☒	☐	Animals and other organisms
☒	☐	Clinical data
☒	☐	Dual use research of concern

Methods

n/a	Involved in the study	
☒	☐	ChIP-seq
☒	☐	Flow cytometry
☒	☐	MRI-based neuroimaging
