# Supplementary material for: Repurposing conformational changes in ANL superfamily enzymes to rapidly generate biosensors for organic and amino acids
Source: Nat Commun. 2023 Oct 21;14:6680. doi: 10.1038/s41467-023-42431-y (PMC10590383; doi:10.1038/s41467-023-42431-y)
Supplement: Supplementary file 4 — Description of Additional Supplementary Files [file 41467_2023_42431_MOESM4_ESM.pdf]

Description of Additional Supplementary Files

Supplementary Data Legends:

Supplementary Data 1: The cpEGFP insertion sites for all GECFINDERs and the detailed library screening data.

Supplementary Data 2: Additional parameters of the GECFINDERs dose-response curves.

Supplementary Data 3: Strains and plasmids used in this study.

Supplementary Data 4: Primers used for cloning in this study
